# Stable pleotropic loci controlling the accumulation of multiple nutritional elements in wheat

**DOI:** 10.1007/s00122-025-04877-0

**Published:** 2025-04-09

**Authors:** Reem Joukhadar, Richard M. Trethowan, Rebecca Thistlethwaite, Matthew J. Hayden, James Stangoulis, Suong Cu, Josquin Tibbits, Hans D. Daetwyler

**Affiliations:** 1 Agriculture Victoria, Centre for AgriBioscience, AgriBio, Bundoora, VIC Australia; 2 School of Life and Environmental Sciences, Plant Breeding Institute, Sydney Institute of Agriculture, The University of Sydney, Narrabri, NSW Australia; 3 School of Life and Environmental Sciences, Plant Breeding Institute, Sydney Institute of Agriculture, The University of Sydney, Cobbitty, NSW Australia; 4 School of Applied Systems Biology, La Trobe University, Bundoora, VIC Australia; 5 College of Science and Engineering, Flinders University, Sturt Road, Bedford Park, South Australia 5042 Australia

## Abstract

Understanding the genetic basis of nutrient accumulation in wheat is crucial for improving its nutritional content and addressing global food security challenges. Here, we identified stable pleiotropic loci controlling the accumulation of 13 nutritional elements in wheat across diverse environments using a large wheat population of 1470 individuals. Our analysis revealed significant variability in SNP-based heritability values across 13 essential elements. Genetic correlations among elements uncovered complex relations, with positive correlations observed within two distinct groups, where calcium (Ca), cobalt (Co), potassium (K), and sodium (Na) formed one group, and copper (Cu), iron (Fe), magnesium (Mg), manganese (Mn), molybdenum (Mo), nickel (Ni), phosphorus (P), and zinc (Zn) formed the other. Negative correlations were observed among elements across both groups. Through MetaGWAS analysis, we identified stable QTL associated with individual elements and elements with high positive correlations. We identified 67 stable QTL across environments that are independent from grain yield, of which 56 were detected using the MetaGWAS analysis indicating their pleiotropic effect on multiple elements. A major QTL on chromosome 7D that can shift the phenotype up to one standard deviation compared to the mean phenotype in the population exhibited differential effects on multiple elements belonging to both groups. Our findings offer novel insights into the genetic architecture of nutrient accumulation in wheat and have practical implications for breeding programmes aimed at enhancing multiple nutrients simultaneously. By targeting stable QTL, breeders can develop wheat varieties with improved nutritional profiles, contributing to global food security and human health.

**Supplementary Information:**

The online version contains supplementary material available at 10.1007/s00122-025-04877-0.

## Introduction

Inadequate intake of crucial vitamins and minerals often leads to a condition known as “hidden hunger” (Sanchez and Swaminathan 2005; Stein and Qaim 2007). The human body relies on several vitamins and minerals, each playing a pivotal role in maintaining overall health (Welch and Graham 2004). These minerals can be divided into two categories: macro-elements (Na, K, Ca, Mg, S, P, and Cl), required in substantial quantities, and microelements (Fe, Zn, Cu, Mn, I, F, B, Se, Mo, Ni, Cr, V, Si, As, Sn, and Co), needed in trace amounts (Welch and Graham 2004). The absence of any of these essential elements from the human diet can result in severe illnesses, developmental challenges in children, decreased resistance to infectious diseases, and overall poor health (Golden 1991; Branca and Ferrari 2002; Alina et al. 2019). Over two billion individuals worldwide are affected by micronutrient deficiencies (Liu et al. 2018). A large proportion of wheat grown in Australia is exported to regions in Asia and the Middle East where micronutrient malnutrition is a widespread issue (Fischer et al. 2014; Kogan et al. 2018; Hunt et al. 2019). Enhancing the concentration of these elements in Australian wheat not only ensures the maintenance of high-quality standards but also presents promising opportunities for improved wheat marketing in these regions.

To enhance grain nutrient content, three distinct approaches can be used. The first strategy is the industrial fortification during wheat flour processing, but this strategy increases milling costs (Ali and Borrill 2020). The second involves agronomic practices such as zinc foliar spray to increase grain nutrient concentrations. For example, Zou et al. (2012) achieved nearly doubled Zn levels with foliar spray, although it proved less effective for Fe (Zhang et al. 2010). Applying nitrogen fertilisers with micronutrients is also an option, but their cost and limited plant absorption make them less environmentally friendly (Gupta et al. 2020). The third and the most effective strategy involves breeding new cultivars with high mineral grain concentrations (Ali and Borrill 2020). Genetic biofortification is more economically viable and sustainable on the long term compared to the industrial and agronomic strategies.

Grain mineral concentrations involve complex traits controlled by multiple genetic pathways, including soil absorption by roots, translocation from root to shoot, and allocation to developing grain (Mori 1999). Environmental factors, soil type, and fertilisation further complicate breeding efforts to improve the concentration of micronutrients elements (Lowe et al. 2020). Therefore, precise genetic dissection of micronutrient element concentration requires multi-environmental data to detect stable quantitative trait loci (QTL) that control the accumulation of multiple elements across wide range of environmental conditions. Genome-wide association studies (GWAS) have been widely adopted to identify genetic variants associated with key traits including mineral accumulation in wheat grains (Gupta et al. 2022). GWAS allows us to detect specific alleles associated with candidate genes that contribute to the accumulation of essential minerals by anslysing the genotypic and phenotypic data in large and diverse wheat populations (Rathan et al. 2022). Several recent studies have utilised GWAS to uncover genetic markers linked to mineral content in wheat, leading to significant markers on various chromosomes and candidate genes involved in metal uptake, transport, and assimilation (Bhatta et al. 2018; Alomari 2017, 2019, 2021; Uffelmann et al. 2021; Hao et al. 2024; Aljabri and El-Soda 2024).

Another strategy to detect QTL is meta genome-wide association or MetaGWAS (Bolormaa et al. 2014). MetaGWAS uses summary statistics instead of actual data over the multivariate linear mixed models (Zhou and Stephens 2014) without having any significant effect on the power of the analysis, assuming the same population size in both analyses (Evangelou and Ioannidis 2013). Therefore, MetaGWA is an optimal solution when data cannot be shared or when unbalanced reference data (i.e. different sets of individuals across different environments) is used leading to increasing the population size and thus, increasing the statistical power of the GWAS (Joukhadar et al. 2021a). However, its application in the field of plant genetics is currently relatively limited (Battenfeld et al. 2018; Zhao et al. 2019; Fikere et al. 2020; Joukhadar et al. 2021a; Singh et al. 2021).

In the present study, we aimed to detect stable QTL across environments with pleotropic effects using MetaGWAS for the concentration of 13 essential nutritional minerals (B, Ca, Co, Cu, Fe, K, Mg, Mn, Mo, Na, Ni, P, and Zn) in wheat grain. Our investigation involved a pre-breeding population comprising 1,470 individuals, all of whom were genotyped using the 90K Illumina Infinium SNP array. These individuals were also subjected to phenotypic evaluation at two different times of sowing (TOS) in Narrabri, New South Wales, for the whole population and in Horsham, Victoria, and Merredin, Western Australia, for a subset of 200 lines.

## Materials and methods

### Plant materials

A total of 1470 spring bread wheat semi-dwarf genotypes were used in this study. These materials are described in Joukhadar et al. (2021b). They were initially chosen based on their potential tolerance to high temperatures. The population included 24 Australian varieties that served as control samples and 1373 genotypes that were developed at the University of Sydney’s Plant Breeding Institute. These genotypes were developed from a wide range of unadapted diversity, including emmer wheat, landraces, and synthetic wheats. Furthermore, the study included 65 wheat genotypes introduced from both the International Maize and Wheat Improvement Centre (CIMMYT) and the International Centre for Agricultural Research in the Dry Areas (ICARDA) and miscellaneous materials including the parents of bi-parental mapping studies and other published findings. All materials had semi-dwarf growth habit and relatively limited phenological variation.

### Phenotypic and genotypic data

The whole population of 1470 was phenotyped with two replicated randomised block designs in two field trials at Narrabri, NSW, one optimally sown (around mid-May) and one late sown (around mid-July) in 2017. A total of 200-line corset were selected based on genetic diversity and genetic estimated breeding values (GEBVs) for grain yield, and these were evaluated in Merredin and Horsham in 2018 in two times of sowing (TOS) each (optimal and late), like Narrabri trials. All six field trials were sown in two replicated randomised complete block experiments. At each site, TOS experiments were conducted side by side. Plot sizes were initially 12 m2, and later trimmed to 8 m2 at harvest.

In the present study, we evaluated thirteen nutritional traits including B, Ca, Co, Cu, Fe, K, Mg, Mn, Mo, Na, Ni, P, and Zn. A total of 250 g grain samples from each harvested plot were used for the test. The nutrient analysis was performed at the Flinders Analytical Laboratory (Flinders University, Australia). After milling, acid digestion was applied on approximately 0.3 g of each sample dried at 80 °C for 4 h, following Wheal et al. (2011). Mineral concentrations were determined using inductively coupled plasma mass spectrometry (ICP-MS 7500x; Agilent, Santa Clara, CA), as described in Palmer and Stangoulis (2018). For control, each digestion batch included a blank and certified reference material (CRM; NIST 1567a wheat flour).

The linear mixed model implemented in ASReml-R (Glimour et al. 2009) was used to calculate the best linear unbiased estimates (BLUEs) for each element within each environment. We used a spatial adjustment that accounted for the field layout, with replications treated as random effects. The model can be represented by the equation: y = *μ* + ***Xf*** + ***Zl*** + *e* (He et al. 2019), where ‘*y*’ represents the phenotypic records, ‘*μ*’ stands for the intercept, ‘*f*’ related to field design including column, row, and replicate (random effects), ‘*l*’ is the genetic value (fixed effect), and ‘e’ represents the residual. The matrices ‘***X***’ and ‘***Z***’ are the incidence matrices for ‘*f*’ and ‘*l*’.

We used the 90 K Infinium SNP array (Wang et al. in 2014) to genotype the population. Genotypes with over 40% missing data were excluded from subsequent analyses. Additionally, SNPs with < 60% call rate and a minor allele frequency (MAF) < 5% were excluded. The final number of SNPs after filtration was equal to 41,666 SNPs. Missing genotypes within the 90K SNPs were imputed using LinkImpute software with the default parameters (Money et al. 2015). The in-silico cross-validation demonstrated an accuracy exceeding 0.99.

### Heritability, genetic correlation, and linkage disequilibrium (LD) analyses

Genetic narrow sense heritability or SNP-based heritability (*h*^2^) for each trait in each environment was calculated using the restricted maximum likelihood (REML) model implemented in MTG2 software (Lee and Van der Werf 2016). The heritability values were averaged across all environments. We also used MTG2 with its bivariate model to estimate the genetic correlations between each pair of traits across all field trials. Values across field trials were averaged to get an overall estimation of the genetic correlations across different nutritional elements. The average genetic correlation values were then used to cluster the nutritional elements into highly correlated groups. Linkage disequilibrium (LD) decay was calculated following Hill and Robertson (1968) by calculating the *r*^*2*^ values between each pair of SNPs located on the same chromosome. These pairwise *r*^*2*^ values were plotted against the distance between each pair of SNPs, and the second-degree Loess smoothing was calculated and plotted. To calculate the critical value for *r*^*2*^, or the background LD, *r*^*2*^ values were calculated for pairs of unlinked SNPs located on different chromosomes. The background LD value was then determined as the 99th quantile of all *r*^*2*^ values between unliked loci. This *r*^*2*^ value was considered as the baseline beyond which we consider that the genetic linkage is the most probable cause for LD (Joukhadar et al. 2017).

### MetaGWAS

The first step when running the MetaGWAS analysis is to run a univariate GWAS analysis for each phenotypic trait (element concentration) in each environment. BLUEs were analysed using the following mixed linear model implemented in GEMMA software (Zhou and Stephens 2012, 2014):

$$y= \mu +X\beta +I\alpha +\varepsilon$$

where $y$ is the BLUEs for all individuals, $\mu$ is the intercept, $\beta$ is the SNP effect, ***X*** is the SNP genotypes, ***I*** is the identity matrix, $\varepsilon$ is the residual, and $\alpha$ represents the random effects derived from the multivariate normal distribution $\alpha \sim MVN(0,\lambda {\tau }^{-1}G)$, in which $\lambda$ is the ratio between variant components, ${\tau }^{-1}$ is the variance of the errors, and ***G*** is the genomic relatedness matrix calculated following Yang et al. (2010).

MetaGWAS analysis was applied for each nutritional element across the six field trials to detect stable QTL for each element, and across multiple elements within each group (based on the genetic correlation) for all field trials to detect stable pleiotropic QTL. We used the model developed by Bolormaa et al. (2014) which calculates a global p-value for each variant considering the SNP effects and standard error calculated for each univariate GWAS analysis. The method starts by calculating a Chi-squared value for each SNP across derived from all univariate analyses as:

$${\chi }_{i}^{2}={t}_{i}^{\prime}{V}^{-1}{t}_{i}$$

where ${t}_{i}$ is the signed *t*-values for the SNP *i* and ${t}_{i}^{\prime}$ is its transpose; ${V}^{-1}$ is the inverse of the correlation matrix among the t-values for all univariate analyses. The *t*-values can be given with the following equation:

$$t=\frac{b}{\text{se}(b)}$$

where *b* is the allelic substitution effect for the SNP calculated in each univariate analysis and se(*b*) is its standard error. The Bonferroni correction was used to declare the significant threshold to correct for multiple testing. Marker trait associations were clustered into different QTL if adjacent loci on the chromosome had high linkage disequilibrium with each other’s with *r*^*2*^ > 0.5.

The genetic correlation analysis revealed two groups; therefore, we run three different MetaGWAS analyses, across group one elements (Meta1), group two elements (Meta2), and across all elements (Meta). These analyses were compared to the single-trait GWAS analysis results for each element within the group. Common and major QTL across both groups were inferred when they were detected in Meta1, Meta2, and Meta analyses. Common and minor QTL across groups were detected only in meta-analysis.

## Results

### Heritability, genetic correlations, and linkage disequilibrium

The average SNP-based heritability values for each element across environments ranged between 0.29 for the accumulation of Co and 0.61 for the accumulation of Ca with an average value of 0.42 over the 13 elements (Table 1). Average genetic correlation across different environments within each element ranged between 0.43 for the accumulation of Na and 0.84 for the accumulation of Ca. Between different elements, the average correlation value ranged between 0.75 for the accumulation between Fe and Zn and -0.45 for the accumulation between K and Mg (Table 1). The elements Ca, Co, K, and Na had positive genetic correlations among each other with an average value of 0.22. Similarly, the elements Cu, Fe, Mg, Mn, Mo, Ni, P, and Zn also had positive genetic correlations among each other with an average value of 0.45. Interestingly, the accumulation of elements across both groups generally had negative correlations with an average value of -0.20. The accumulation of B had a generally low positive genetic correlation with the elements belonging to group two and low negative correlations with three out of four elements from group one. Therefore, B was included in the second group for the multi-trait/multi-environmental MetaGWAS analysis.

**Table 1:**
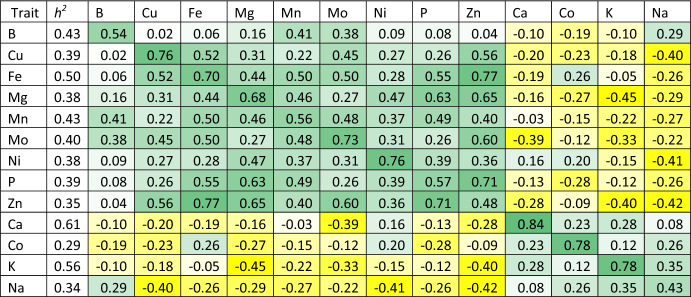
SNP-based heritability and average genetic correlation for each pair of elements across all environments. Colours are scaled from the lowest *r* in yellow to the highest *r* in green

Comparing the average genetic correlation for each element across the environments to its average genetic correlation with other elements revealed several cases, in which the environment had more influence on an element across trials than its relatedness to other elements. The concentration of Zn showed the largest number of superior across element genetic correlations with five elements having a higher correlation with Zn compared to the correlation of Zn trials (Table 1). Across environments, Zn had an average genetic correlation of 0.48 which is lower than its correlations with Cu, Fe, Mg, Mo, and P which ranged between 0.56 and 0.77. The same applied to the accumulation of P which had high correlations with Zn and Mg, and for Fe which had higher correlation with Zn (Table 1). LD decay analysis showed that an extended decay and large LD blocks in the studied population. The background LD was equal to 0.176 which intersect with around 7 Mb on the LD decay smoothing line (Figure S1), indicating that the average size of LD blocks in our germplasm was equal to 7 Mb.

### QTL detected with MetaGWAS

The MetaGWAS analysis was conducted on three levels. First, for the accumulation of each element across environments was analysed independently from the remaining elements to detect stable QTL for the accumulation of the element across all environments. Second, across environments/group of elements with high positive correlations with each other were analysed to detect stable QTL with pleotropic effects within the group (Termed Meta1 for the first cluster of elements and Meta2 for the second cluster of elements). Third, across all elements in all environments (termed “Meta”) to confirm QTL detected with both Meta1 and Meta2, as well as detecting other minor genes that were not reported in the previous analyses.

For elements belonging to cluster one (Ca, Co, K, and Na), each element had a single QTL except for K which had two different QTL (Table 2). The Na QTL was located on the long arm of the chromosome 1B (~ 689.8mb), while the Co QTL was located on the long arm of chromosome 7D. Three SNPs with high LD (*r*^*2*^ > 0.5) were associated with this QTL with large interval distances among them from 268.4 to 453.0 Mb but a genetic distance between 134.4 and 139.1 cM. The accumulation of Ca and K was associated with a QTL on the short arm of chromosome 7D around 60.2 Mb. The accumulation of K had another QTL on the long arm of chromosome 5B around 509.8 Mb. Interestingly, the multi-trait analysis (Meta1) showed significant association for at least one SNP for all four QTL associated with the accumulation of group one elements. This is consistent with the single-trait analysis, in which the accumulation of most elements had − log_10_(*p*) value > 1 (Table 2) indicating a minor effect for these QTL to other elements within the group.

**Table 2:**
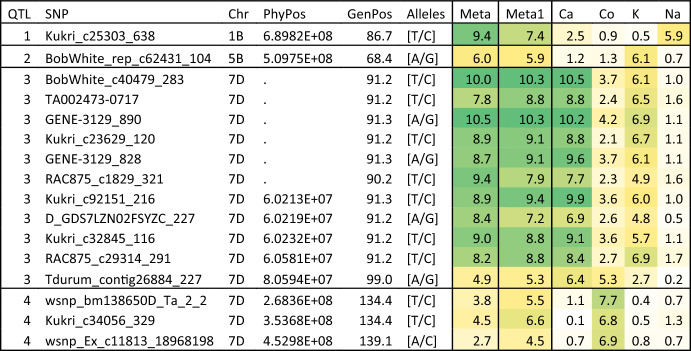
QTL detected by applying MetaGWAS analysis on the group one elements (Ca, Co, K, and Na; Meta1), or all elements (Meta) that were also detected for the accumulation of some of the group one elements

Colours are scaled from the lowest -log_10_(*p*) in white to the highest -log_10_(*p*) in green

PhyPos: physical position; GenPos: genetic position

A total of 25 QTL were detected for the accumulation of the elements belonging to the second group which are B, Cu, Fe, Mg, Mn, Mo, Ni, P, and Zn (Table 3). The accumulation of these elements had 2, 8, 6, 3, 2, 4, 0, 1, and 4 associated QTL, respectively. The accumulation of Mg had three QTL on chromosomes 3A, 3B, and 5B. The last one was also associated with the accumulation of P and Mo. Three other QTL were associated with Mo accumulation, one on chromosome 6B, and two on chromosome 7D. The two QTL on chromosome 7D were also associated with the B accumulation (Table 3). The accumulation of Zn had two different QTL on chromosome 5A plus one QTL on chromosome 6A and another on chromosome 3B. The last QTL was also associated with the Mg accumulation. The accumulation of Mn had two QTL on 4A and 6A, while Fe accumulation had six QTL on chromosomes 3A, 3D, 4B, 5D, and 6D. The accumulation of Cu had the largest number of associations with 8 QTL on chromosomes 1A, 1B, 1D, 2B, 3A, 5D, and 6A. Interestingly, 14 out of the 25 QTL associated with the accumulation of group two elements were detected with the across element multi-trait analysis (Meta2, Table 3). Similar to the case of Meta1 QTL, these QTL also showed − log_10_(p) value > 1 for multiple elements within the group.

**Table 3:**
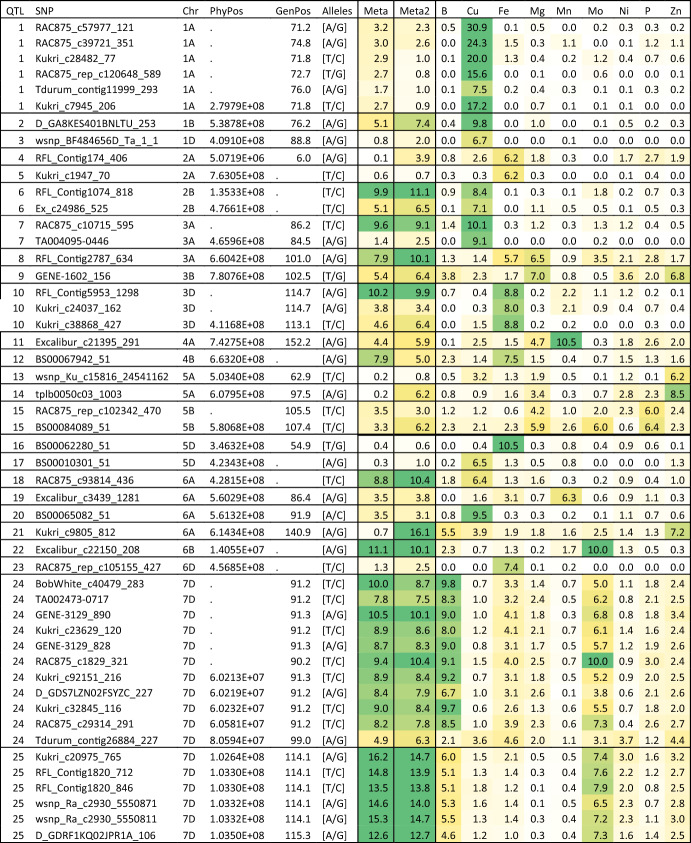
QTL detected by applying MetaGWAS analysis on the group two elements (B, Cu, Fe, Mg, Mn, Mo, Ni, P, and Zn; Meta2), or all elements (Meta) that were also detected for the accumulation of some of the group two elements

Colours are scaled from the lowest -log_10_(*p*) in white to the highest -log_10_(*p*) in green

PhyPos: physical position; GenPos: genetic position

**Fig. 1:**
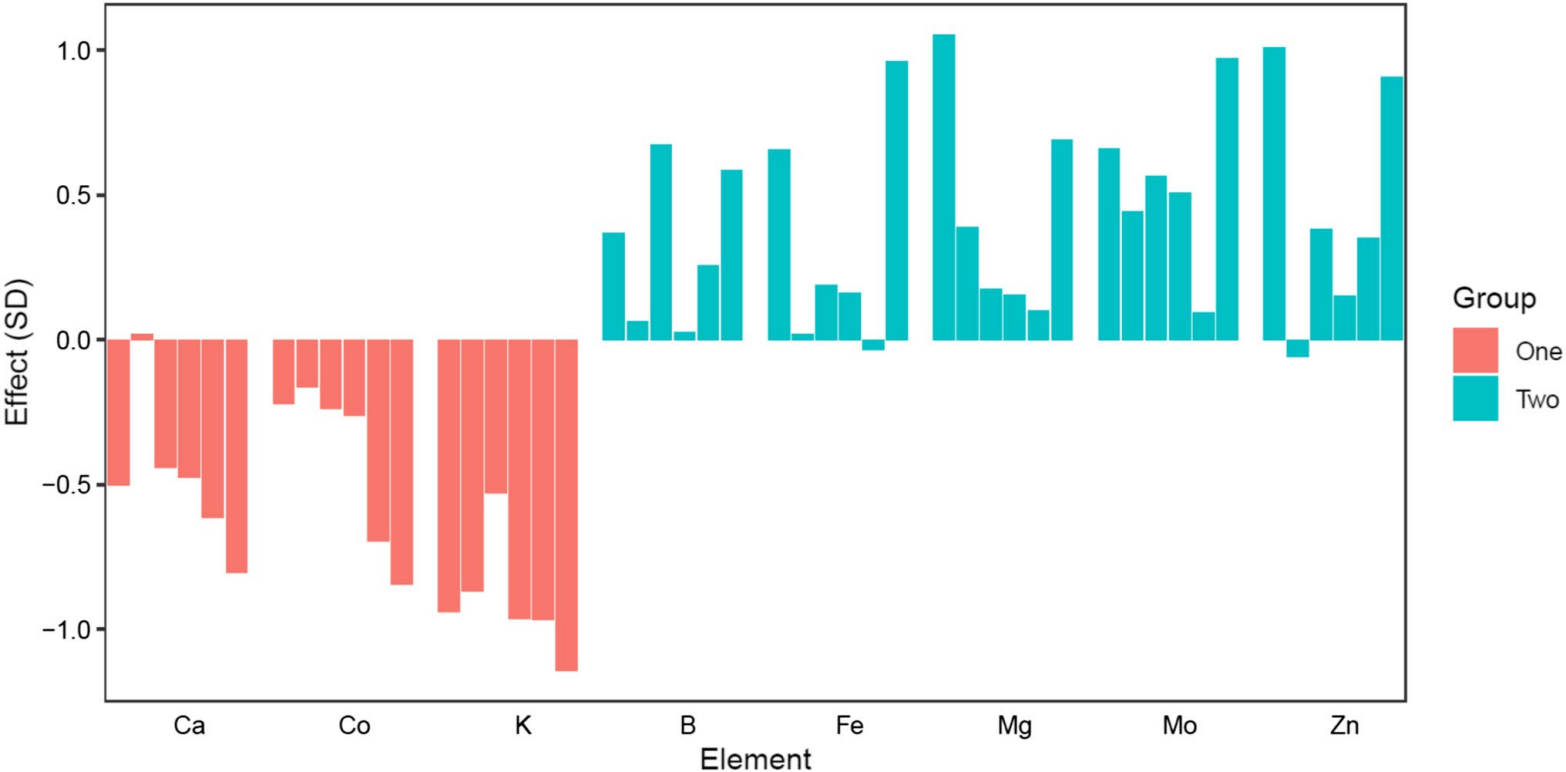
Allelic effect of the SNP GENE-3129_828 on chromosome 7D which showed association with MetaGWAS analysis for both group one and group two elements. The effect was measured as the number of phenotypic standard deviation between the genotypes that carry the allele “G” compared to the genotypes that carry the allele “A”. Environments for each element were in the following order: H1, H2, M1, M2, N1, and N2

Comparing the results between Meta1 and Meta2, there were only a single common QTL on chromosome 7D at 60.2 Mb that was associated with both analyses (Tables 2, 3). This QTL had major effect on several elements belonging to different groups with − log_10_(*p*) value = 10.5. However, the effects of this QTL had differential magnitudes between group one and group two elements. In other words, the allele that increases the accumulation of group one elements decreased the accumulation of group two elements (Fig. 1). This QTL is collocated with a region that involved two transmembrane proteins, which could be responsible for the transport of nutritional elements to the grains. The first gene *LOC123166635* (601, 963, 309..601, 965, 386) which encodes heptahelical transmembrane protein ADIPOR2-like, while the other gene is *LOC123163701* (603, 067, 213..603, 068, 753) which was annotated as protein NRT1/PTR FAMILY 5.1-like.

There were another 24 QTL that showed significant association with the across element MetaGWAS analysis without passing the significant threshold for the accumulation of any individual element (Table 4), indicating a minor consistent small effect across several elements. Of these, 23 QTL were associated with the accumulation of group two elements while only one QTL on chromosome 2B was associated with the accumulation of group one elements. The group two QTL were distributed on chromosomes 1B, 2A, 2B, 2D, 3A, 3B, 3D, 4A, 4B, 5A, 6B, 7A, and 7B. The largest number of QTL was located on the chromosomes 3A and 6B with each having four different QTL. Another 14 QTL were significantly associated only when using all elements in all environments, analysis “Meta” (Table 4). Interestingly, all element accumulation related QTL detected in the present study were independent from the grain yield QTL detected in our previous MetaGWAS study (Joukhadar et al. 2021a).

**Table 4:**
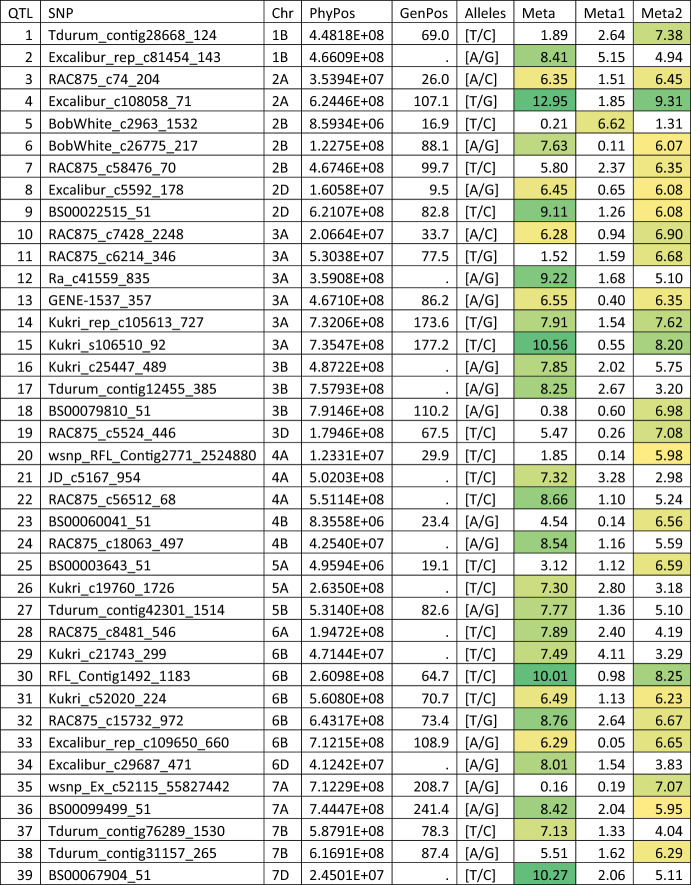
QTL detected by applying MetaGWAS analysis on both group one (Meta1), group two elements (Meta2), or all elements (Meta) that were not detected for the accumulation of any element independently. Colours are scaled from the lowest -log_10_(*p*) in white to the highest -log_10_(*p*) in green

PhyPos: physical position; GenPos: genetic position

## Discussion

Increasing grain yield in wheat has led to a reduction in the concentration of essential nutritional elements in the grains due to the higher accumulation of starch in a phenomenon named the dilution effect (Amiri et al. 2015). This phenomenon can be attributed to the plant’s allocation of resources—when the selection emphasis is on increasing grain size and number, the plant may divert nutrients away from other crucial metabolic processes, resulting in lower nutrient concentrations (Gupta et al. 2020). This is a serious issue especially in developing countries where malnutrition is a common problem, and wheat is a major source of calories on the population level (Welch and Graham 2004; White and Broadley 2009). It highlights the importance of adopting a balanced approach to agricultural advancements, where efforts to boost yield are coupled with strategies to maintain or enhance the nutritional quality of crops (Trethowan et al. 2005). This would ensure that increasing grain yield doesn’t come at the cost of diminished nutritional value that has a negative impact on human health (Ortiz-Monasterio and Graham 2000). The present study aimed to unravel the genetic basis of nutrient accumulation in wheat by investigating genetic correlations and identifying environmentally stable pleiotropic loci through MetaGWAS analysis. Our findings provide valuable insights into the complex genetic control of nutrient accumulation and have practical implications for wheat breeding and crop improvement. Moreover, one of the QTLs detected in the present study overlapped with a grain yield QTL reported on the same population in Joukhadar et al. (2021a), despite the negative correlation between grain yield and group one elements (Joukhadar et al. 2021b) indicating independent pathways for accumulating elements and starch.

Substantial variation in SNP-based heritability values across the 13 nutritional elements was observed. These heritability estimates offer guidance for wheat breeding programmes, emphasising elements with higher heritability as more amenable to genetic improvement (Schmidt et al. 2019). Genetic correlations among elements unveiled complex relationships between nutrient accumulations. Positive genetic correlations were observed within two distinct groups of elements: Ca, Co, K, and Na formed one group, while B, Cu, Fe, Mg, Mn, Mo, Ni, P, and Zn comprised the other. These positive correlations suggest coordinated genetic control mechanisms for elements within each group, which can aid in the design of breeding strategies targeting multiple nutrients simultaneously. Negative genetic correlations were common when examining the accumulation of elements across both groups, suggesting potential trade-offs in nutrient allocation (Neyhart et al. 2019). Therefore, our MetaGWAS study was designed to split the elements into two groups based on their genetic correlation structure. Such findings highlight the complexity of nutrient accumulation in wheat and emphasise the need for careful consideration in breeding programmes that aim to enhance multiple nutrients simultaneously (Morgounov et al. 2007). Interestingly, the two most important elements for global nutritional security (Fe and Zn) are positively correlated, streamlining the simultaneous improvement for both (Manickavelu et al. 2017; Bhatta et al. 2018; Alomari et al. 2019).

In previous research on the same data (Joukhadar et al. 2021b), we also investigated the SNP-based heritability and genetic correlations across the elements. However, in the previous study, environment was treated as a fixed effect, therefore, a single heritability value per element and a single overall genetic correlation value for each pair of elements was calculated. Here, we ran independent tests for each environment and averaged the heritability and genetic correlation values. The advantage achieved here was the ability to compare the genetic correlation for each element across environments to that obtained when compared to other elements. The heritability values in the present study were slightly less than double that reported in the previous study. This is expected given that the SNP-based heritability represents the shared genetic variance across different environments which should theoretically be affected when increasing the number of environments (Lee and van der Werf 2016; Yang et al. 2010). However, the genetic correlation values were comparable in both studies. Several elements, specifically Zn, P, and Fe, showed higher across element average genetic correlations compared to the within-element across environment average genetic correlation. This indicated that environment has a larger influence on the variation of the element itself than its genetic relation (or the shared genetic variance) with other elements.

The application of MetaGWAS in crop genetics has become more prevalent in recent years. Meta-analytical methods were first proposed to combine data across different partners when raw data cannot be shared (Winkler et al. 2014). When applied to multi-trait or multi-environment crop breeding data, they offer a simple solution to analyse unbalanced data given that designing balanced breeding programmes is often impractical (Joukhadar et al. 2021b). The significance of a QTL detected with MetaGWAS increases for QTL that are expressed in larger number of individual univariate GWAS analyses. In other words, the effect of the QTL should be correlated across significant number of involved traits/environments. Group two had more QTL compared to group one with 37 and 5 QTL, respectively. This was because group two had a larger number of elements that had higher genetic correlations among them compared to group one, leading to larger number of loci affecting all trials/traits. However, many of these QTL were revealed in the full analysis (Meta) that involved all elements in all environments regardless of the correlation structure. This indicates they still have an effect, although small, on the elements belonging to the other group. It was possible to detect this small effect using the MetaGWAS because this approach increases the population size which is critical to detect minor genes. The allelic effect for the major 7D QTL that was detected in both groups was opposite across the groups. This comes in concordance with the negative genetic correlation across both groups. Fine mapping this QTL and analysing the function of its gene will be important in the future given its complex interaction and large effect on different elements.

Several of the QTL identified in this study have been previously reported. Out of the five QTL detected for group one elements, the QTL for K appears to co-locate with a previously reported QTL on the long arm of chromosome 5B (Alomari et al. 2021). The remaining QTL seem to be novel. For group two QTL, one reported QTL associated with Mn accumulation (Wang et al. 2021) was in the same position of our Meta-QTL on chromosome 1B at 538.78 Mb. Another Meta-QTL on chromosome 3A at 20.66 Mb (Table 4) was previously reported to be associated with Mg (Hao et al. 2024) and Cu (Bhatta et al. 2018). The Meta-QTL located at 614.3 Mb on chromosome 6A was previously reported to be associated with Zn (Zhou et al. 2020). Another nine QTL were also previously reported for either Fe, Zn or both, which are the most studied elements in published literature. Alomari et al. (2019) detected a QTL for Fe on chromosome 2A which is very close to our Fe QTL around 763.05 Mb, while Hao et al. (2024) detected another Fe QTL near our 5B QTL at 580.07 Mb. Similarly, Zhou et al. (2020) detected two Zn QTL in the same positions of our Zn QTL on 3B (780.76 Mb) and our Meta-QTL on 6B (712.15 Mb). Another two Zn QTL were previously reported on 7D at 102.64 Mb (Hao et al. 2024) and 2B at 467.46 Mb (Cu et al. 2020). Three other QTL for both Fe and Zn reported in the present study were also reported for the same traits on chromosome 2A at 5.07 Mb (Singh et al. 2021) and 624.46 Mb (Cu et al. 2020), as well as chromosome 3D at 179.46 Mb (Hao et al. 2024).

Out of the detected QTL, one on chromosome 7D (~ 602 Mb) showed highly significant association and effect across wide range of elements. However, its effect is contradictory across elements belonging to both clusters of elements (Fig. 1). There were two genes flanking the most associated SNPs that are involved in transmembrane activity. The first one is *LOC123166635* which encodes heptahelical transmembrane proteins ADIPOR2-like. These proteins are often involved in signal transduction and can act as receptors for various molecules (Hsieh et al. 2005). ADIPOR2-like proteins could potentially be involved in nutrient signalling and homeostasis, influencing the uptake and distribution of micronutrients. The other one is *LOC123163701* which was annotated as NRT1/PTR FAMILY 5.1-like. NRT1/PTR proteins play a crucial role in the transport of nitrate and peptides across cell membranes (Chiba et al. 2015; Deng et al. 2023). These proteins are involved in nitrate uptake and assimilation, which are essential processes for plant growth and development. NRT1/PTR FAMILY 5.1-like proteins could be involved in regulating nitrate absorption and distribution, impacting the overall nitrogen use efficiency of the plant. This can influence the nutritional quality of the grains, including the levels of various micronutrients. However, the specific role of these proteins in wheat grains would require further research and experimental validation. Moreover, the 90K SNPs detected here can be useful to develop KASP maker for easier implementation for marker assisted selection. Targeting these specific candidate genes allows researchers to get more insights into the mechanisms responsible for nutrient accumulation in wheat. Understanding these underlying genetic processes enables the development of wheat varieties with enhanced nutritional profiles. Insights gained from studying these genes can inform breeding programmes, making them more efficient and effective in enhancing multiple nutrient levels simultaneously.

## Conclusion

In conclusion, our study unveiled new insights into the genetic architecture of nutrient accumulation in wheat. The identification of pleiotropic QTL underscores the potential for coordinated breeding to enhance multiple nutrients simultaneously. The intricate genetic relationships among nutrients highlight the complexity of nutrient allocation in wheat. The differential effects of shared QTL between element groups raises intriguing questions about the underlying genetic mechanisms and trade-offs that may exist. Practically, our findings hold promise for wheat breeding programmes aimed at improving the nutritional content of wheat grain. By leveraging these findings, wheat breeders can work towards developing wheat varieties with improved nutritional content, addressing critical challenges in global food security and human health. This research contributes to ongoing efforts to enhance the nutritional value of staple crops and underscores the importance of genomics in modern agriculture.

## Supplementary Information

Below is the link to the electronic supplementary material.

Supplementary file1 (PDF 361 KB)

## Data Availability

The data will be available with reasonable request from the correspondence authors.

## Abbreviations

GWAS: Genome-wide association study
MAF: Minor allele frequency
QTL: Quantitative trait loci
REML: Restricted maximum likelihood
TOS: Time of sowing
