# Supplementary figures and images for: Stable pleotropic loci controlling the accumulation of multiple nutritional elements in wheat
Source: Theor Appl Genet. 2025 Apr 9;138(5):95. doi: 10.1007/s00122-025-04877-0 (PMC11982167; doi:10.1007/s00122-025-04877-0)

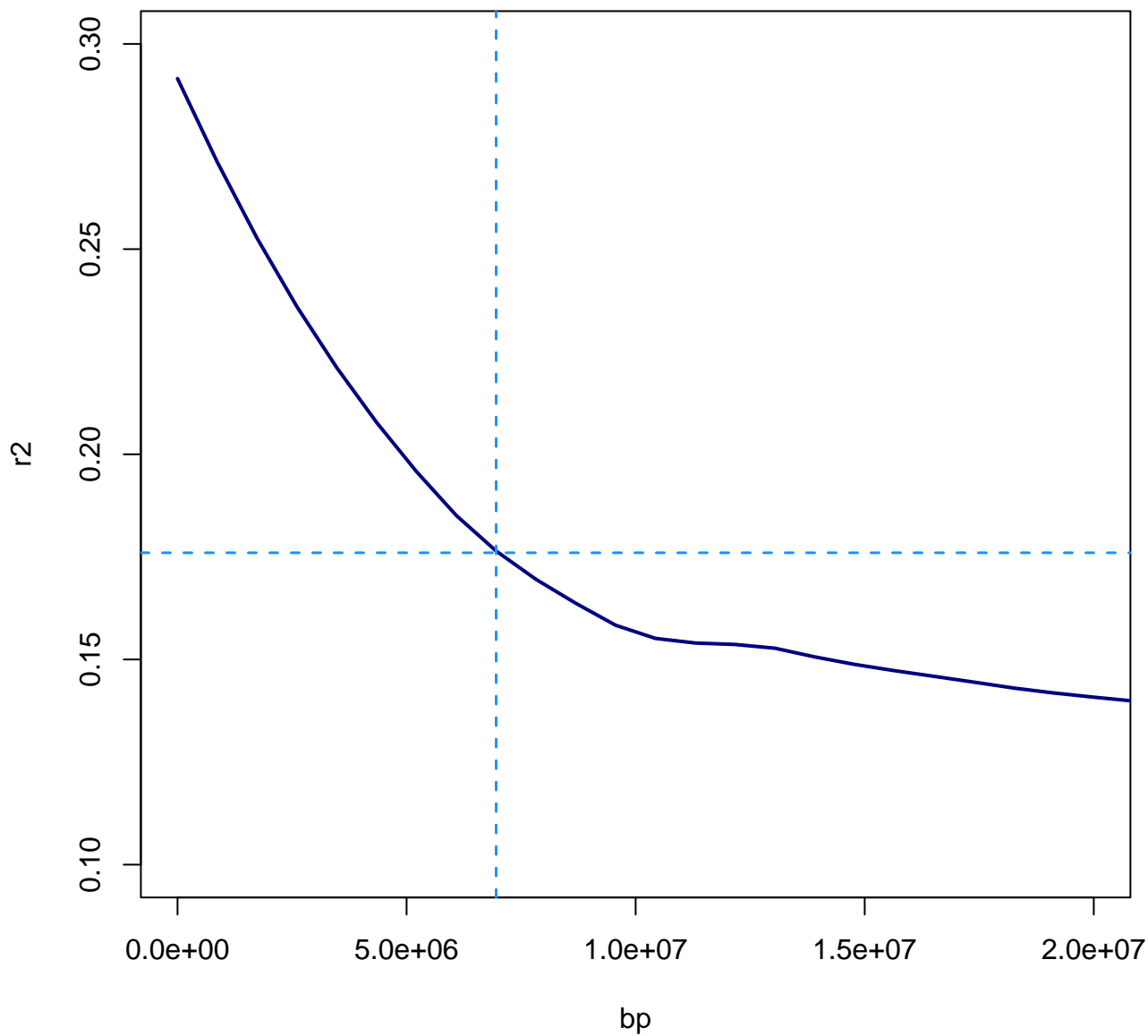

Supplement: Supplementary file 1 — Supplementary file1 (PDF 361 KB) [file 122_2025_4877_MOESM1_ESM.pdf]
